# Trace and major elements distribution in coastal sediment cores from Lagos Lagoon, Nigeria

**DOI:** 10.1016/j.dib.2018.05.030

**Published:** 2018-05-19

**Authors:** Simeon I. Ekett, Omowunmi H. Fred-Ahmadu, Adebusayo E. Adedapo, Nsikak U. Benson

**Affiliations:** Analytical and Environmental Chemistry Unit, Department of Chemistry, Covenant University, Km 10 Idiroko Road, Ogun State, Nigeria

## Abstract

Heavy metals contamination in aquatic ecosystems has been a major concern, however, no information is available about the concentrations, depth distributions and ecological risks of heavy metals in sediments cores from microtidal lagoonal ecosystem in Lagos, Nigeria. Four sediment cores were collected using a 50 cm × 3.5 cm Wildco® hand corer. Elemental determinations were carried out using the Microwave Plasma Atomic Emission Spectrometry (4200 MP-AES) after extraction. The concentrations of heavy metals in the sediment cores indicated enhanced degree of contamination influenced by anthropogenic discharges especially industrial effluents. Vertical depth distributions indicated varied depositional periods largely controlled by increasing anthropogenic land-based activities. The remarkable metal pollution of core sediments raises the concern potential sources of metals to the lagoonal ecosystem and ecological risks to the biota, humans, and the environment. The results of elemental concentrations should be considered as baseline data for heavy metals in sediments in the region.

**Specifications Table**

**Table:** 

Subject area	Environmental Sciences
More specific subject area	Environmental Chemistry
Type of data	Table, text file, graph, figure
How data was acquired	Total digestion and Microwave Plasma Atomic Emission Spectrometry (Agilent 4200 MP-AES)
Data format	Raw data, analyzed.
Experimental factors	Sediment cores were collected using a 50 cm × 3.5 cm Wildco® hand corer. 0.5 g of air-dried sediment samples were accurately weighed and passed through 2 mm sieve. 20 mL of digestion mixture (1:2:2 Perchloric acid: Nitric acid: Sulphuric acid) was used for digesting each sediment sample in the fume hood.
Experimental features	Major elemental analysis using MP-AES.
Data source location	Lagos lagoon, Nigeria, Gulf of Guinea
Data accessibility	Data available within the article.

**Value of the data**

• Baseline depositional distributions of heavy metals in core sediments.
• Geochemical and contamination profiles of elements of lagoonal ecosystem.
• Determine to establish relationship between anthropogenic land-based activities and degree of metal pollution.

## 1 Data

**Fig. 1:**
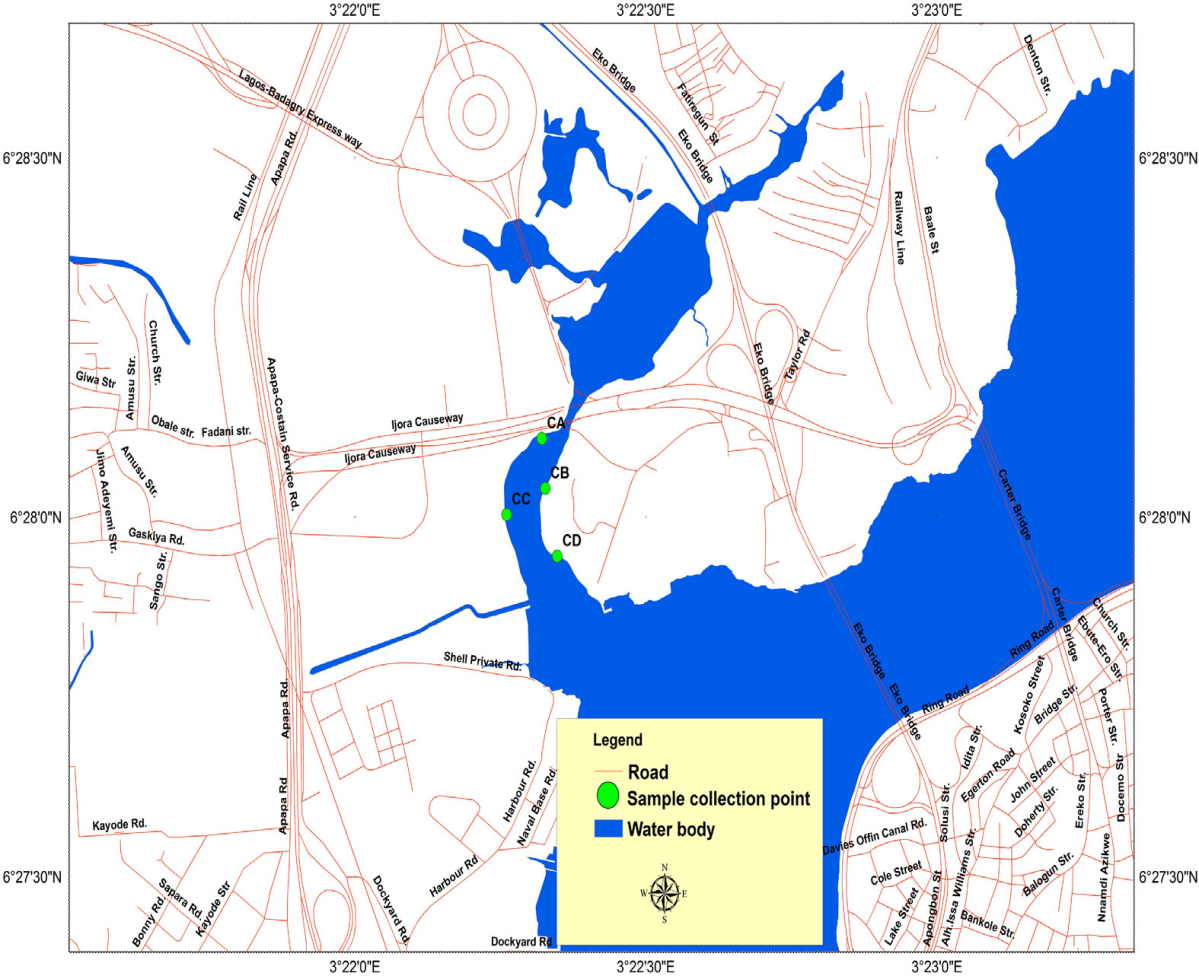
Map of study area showing sediment core sampling locations.

The sampling locations were chosen from the microtidal Lagos lagoon, Nigeria, Gulf of Guinea (Fig. 1). Table 1, Table 2, Table 3, Table 4, Table 5, Table 6, Table 7, Table 8 is representing the concentration (mg/kg) and percentage relative standard deviation of heavy metals in core sediments of the lagoonal estuarine ecosystem. Table 9 indicates the computed degree of contamination using hazard quotient and modified hazard quotient.

**Table 1:** Concentration (mg/kg) of elements in core sediment samples from site A.

**Sample Label**	**Al**	**Ba**	**Ca**	**Cd**	**Co**	**Cr**	**Cu**	**Fe**	**K**	**Mg**	**Mn**	**Na**	**Ni**	**Pb**	**Sr**	**V**	**Zn**
Core A.1	120	0.11	58.1	0.39	0.10	0.20	0.21	146	15.0	32.9	2.47	45.8	0.03	0.45	0.45	0.22	1.11
Core A.2	142	0.11	35.1	0.77	0.11	0.22	0.48	156	17.1	34.3	2.31	76.3	0.05	0.68	0.40	0.16	2.10
Core A.3	225	0.04	54.9	0.82	0.17	0.35	0.69	220	23.8	49.1	2.93	111	0.07	1.11	0.60	0.33	2.73
Core A.4	242	0.06	62.0	0.94	0.18	0.40	0.79	228	24.2	50.1	3.08	93.6	0.07	1.51	0.62	0.37	2.90
Core A.5	483	0.07	113	2.01	0.47	0.88	1.64	477	43.7	97.9	5.18	212	0.23	3.08	1.28	0.95	5.80
Core A.6	228	0.05	43.1	0.94	0.17	0.41	0.82	219	23.6	47.6	2.79	101	0.08	1.15	0.55	0.25	3.37
Core A.7	116	0.07	65.8	0.64	0.11	0.28	0.65	168	16.0	39.8	2.86	70.3	0.07	0.91	0.66	0.18	2.93
Core A.8	119	0.08	96.1	0.82	0.05	0.21	0.50	150	15.8	35.0	2.57	74.6	0.03	0.36	1.70	0.19	2.29
Core A.9	317	0.04	114	1.55	0.28	0.51	0.98	334	33.0	78.0	4.63	163	0.10	1.45	1.43	0.55	2.95
Core A.10	226	0.06	39.9	1.14	0.19	0.36	0.57	266	25.4	55.5	3.84	128	0.06	1.19	0.65	0.41	2.44
Core A.11	88.9	0.12	51.5	0.51	0.03	0.15	0.24	123	15.3	35.7	2.60	84.8	0.02	0.18	0.87	0.17	1.11
Core A.12	177	0.08	47.3	0.66	0.13	0.27	0.65	175	18.6	37.2	2.00	86.0	0.06	0.79	0.50	0.25	2.97
Core A.13	154	0.07	50.9	0.35	0.13	0.24	0.61	155	16.1	31.5	2.34	37.2	0.06	0.71	0.55	0.29	2.59
Core A.14	171	0.06	40.5	0.62	0.12	0.25	0.71	201	18.2	39.8	2.30	92.9	0.06	0.97	0.50	0.30	2.99
Core A.15	225	0.07	43.6	0.80	0.17	0.31	0.65	209	21.4	41.4	2.65	96.8	0.07	1.00	0.51	0.33	2.90
Core A.16	213	0.05	65.8	0.85	0.15	0.30	0.66	184	18.4	39.8	2.80	85.7	0.07	0.76	0.68	0.19	2.64
Core A.17	170	0.07	40.1	0.57	0.11	0.27	0.58	151	17.2	34.8	2.31	70.3	0.06	0.93	0.43	0.24	2.19
Core A.18	169	0.08	49.1	0.54	0.12	0.28	0.62	180	18.5	41.3	2.56	84.7	0.05	0.84	0.52	0.24	2.49
Core A.19	279	0.05	66.6	1.28	0.27	0.51	1.17	365	32.2	69.9	4.21	150	0.11	2.12	0.83	0.62	4.10
Core A.20	188	0.06	51.6	0.61	0.16	0.32	0.77	216	20.3	45.1	3.02	82.0	0.14	1.32	0.59	0.35	2.83
Core A.21	179	0.07	51.9	0.93	0.15	0.32	0.67	213	20.2	44.1	3.04	76.1	0.06	1.24	0.54	0.25	2.31
Core A.22	169	0.10	33.9	0.76	0.09	0.23	0.49	191	18.2	34.2	2.33	65.4	0.03	0.71	0.36	0.07	1.57
Core A.23	185	0.10	53.6	0.61	0.14	0.29	0.38	169	19.0	39.3	2.95	83.1	0.05	0.71	0.46	0.26	1.88
Core A.24	241	0.05	54.6	0.93	0.25	0.34	0.43	260	27.2	56.0	3.58	132	0.06	1.10	0.58	0.44	1.79
Core A.25	191	0.01	42.5	0.58	0.19	0.26	0.34	181	19.8	40.0	2.72	81.7	0.05	0.87	0.44	0.26	1.53

**Table 2:** Calculated % relative standard deviation of concentrations in core sediments from site A.

**Sample Label**	**Al**	**Ba**	**Ca**	**Cd**	**Co**	**Cr**	**Cu**	**Fe**	**K**	**Mg**	**Mn**	**Na**	**Ni**	**Pb**	**Sr**	**V**	**Zn**
A1.1	2.03	0.08	0.23	0.20	0.04	0.07	0.04	1.36	1.36	0.85	0.35	2.07	0.01	0.17	0.13	0.08	0.85
A2.1	2.81	0.07	0.42	0.52	0.09	0.03	0.09	1.42	1.56	0.34	0.30	2.61	0.01	0.22	0.19	0.02	2.00
A3.1	1.96	0.01	1.45	0.24	0.02	0.04	0.56	1.05	0.76	0.68	1.19	1.22	0.03	0.87	0.17	0.09	0.70
A4.1	2.07	0.03	0.62	0.31	0.17	0.10	0.39	1.19	0.31	0.80	1.92	2.11	0.01	1.02	0.18	0.05	1.35
A5.1	1.03	0.03	1.34	1.27	0.08	0.23	0.85	2.69	0.39	1.06	1.41	0.63	0.08	0.57	0.70	0.28	1.23
A6.1	1.96	0.01	2.71	0.25	0.04	0.38	0.67	1.05	1.16	2.05	1.72	1.34	0.02	0.18	0.11	0.06	2.16
A7.1	2.18	0.02	1.77	0.18	0.03	0.02	0.56	0.36	0.94	0.79	0.61	4.43	0.01	0.14	0.14	0.14	1.79
A8.1	2.38	0.05	2.07	0.33	0.01	0.03	0.16	0.75	0.70	0.68	0.58	3.44	0.02	0.07	0.56	0.05	2.01
A9.1	2.52	0.01	1.63	0.95	0.01	0.12	0.69	1.33	1.62	1.55	1.43	1.57	0.01	0.38	0.17	0.43	2.06
A10.1	0.66	0.05	0.21	0.69	0.01	0.15	0.53	2.31	0.95	0.68	3.33	4.07	0.01	0.13	0.11	0.28	2.34
A11.1	0.76	0.33	0.18	0.12	0.02	0.08	0.15	1.46	1.32	0.50	0.79	0.88	0.01	0.05	0.10	0.14	0.21
A12.1	2.22	0.13	1.71	0.18	0.06	0.18	0.13	1.00	1.47	0.61	1.26	3.09	0.02	0.20	0.09	0.16	1.86
A13.1	1.35	0.09	1.21	0.20	0.02	0.06	0.12	0.43	1.70	0.49	0.53	2.10	0.03	0.15	0.10	0.14	1.25
A14.1	1.34	0.72	0.88	0.17	0.03	0.20	0.47	1.34	0.86	1.38	1.17	0.82	0.03	0.12	0.18	0.05	0.86
A15.1	2.00	0.03	0.86	0.50	0.03	0.20	0.40	0.39	1.13	0.70	1.53	0.49	0.02	0.12	0.11	0.12	1.74
A16.1	2.05	0.12	1.05	0.63	0.03	0.27	0.11	1.44	0.90	2.29	0.51	0.14	0.02	0.28	0.06	0.06	1.68
A17.1	0.99	0.35	2.25	0.20	0.02	0.11	0.23	0.96	1.34	2.20	1.31	3.64	0.01	0.29	0.12	0.15	0.23
A18.1	0.94	0.05	1.21	0.21	0.04	0.07	0.14	1.30	0.64	2.32	1.64	3.40	0.03	0.80	0.17	0.07	2.27
A19.1	1.09	0.53	1.08	0.44	0.05	0.25	0.50	0.56	1.41	1.28	1.24	2.71	0.01	0.60	0.10	0.06	2.09
A20.1	0.21	0.02	2.34	0.04	0.02	0.12	0.57	2.52	1.38	1.61	0.98	0.15	0.05	0.20	0.07	0.11	2.01
A21.1	0.89	0.21	0.74	0.17	0.11	0.18	0.11	2.11	1.01	0.22	0.95	1.35	0.03	0.11	0.15	0.13	2.14
A22.1	0.88	0.38	1.37	0.49	0.07	0.20	0.10	0.87	0.33	1.59	1.03	0.39	0.01	0.16	0.14	0.01	1.50
A23.1	1.84	0.02	0.90	0.09	0.04	0.10	0.14	0.47	3.65	1.62	0.64	2.86	0.04	0.07	0.38	0.08	0.24
A24.1	1.27	0.01	1.28	0.05	0.01	0.16	0.08	0.43	0.16	0.70	0.91	0.96	0.03	0.12	0.15	0.05	1.28
A25.1	0.65	0.01	0.48	0.51	0.02	0.03	0.10	1.31	1.40	0.71	0.22	0.76	0.01	0.19	0.07	0.01	0.19

**Table 3:** Concentration (mg/kg) of elements in core sediment samples from site B.

**Sample Label**	**Al**	**Ba**	**Ca**	**Cd**	**Co**	**Cr**	**Cu**	**Fe**	**K**	**Mg**	**Mn**	**Na**	**Ni**	**Pb**	**Sr**	**V**	**Zn**
Core B.1	172	0.09	28.6	0.70	0.17	0.24	0.35	193	18.5	36.2	2.35	62.3	0.05	0.81	0.37	0.28	1.71
Core B.2	245	0.06	66.1	1.03	0.22	0.34	0.50	241	24.5	49.3	2.98	82.7	0.08	1.28	0.72	0.20	1.93
Core B.3	192	0.07	42.7	1.00	0.17	0.25	0.35	203	21.5	38.8	2.58	64.8	0.06	0.86	0.49	0.08	1.29
Core B.4	262	0.04	30.1	0.88	0.24	0.32	0.27	210	23.7	40.8	2.76	67.8	0.07	0.98	0.36	0.40	1.36
Core B.5	333	0.03	50.5	1.18	0.35	0.48	0.45	285	29.8	53.9	3.20	92.6	0.11	1.61	0.60	0.40	1.71
Core B.6	404	0.01	53.3	1.46	0.38	0.52	0.46	334	31.8	60.6	3.88	97.7	0.13	1.30	0.66	0.58	1.89
Core B.7	259	0.07	40.9	1.05	0.27	0.34	0.37	333	29.1	54.3	3.44	91.7	0.05	1.28	0.49	0.59	1.58
Core B.8	378	0.04	39.4	1.29	0.33	0.48	0.33	287	29.1	52.6	3.21	83.6	0.10	1.20	0.48	0.49	1.42
Core B.9	231	0.08	32.5	0.53	0.23	0.36	0.31	230	23.3	43.7	3.02	67.4	0.06	1.08	0.40	0.38	1.46
Core B.10	260	0.06	39.0	1.00	0.26	0.36	0.30	237	25.8	47.0	2.97	73.5	0.08	1.06	0.46	0.30	1.30
CoreB.11	345	0.05	45.4	1.19	0.33	0.46	0.42	305	31.6	57.7	3.67	98.9	0.10	1.30	0.59	0.55	1.61
Core B.12	186	0.07	33.2	0.52	0.19	0.27	0.22	180	18.9	35.8	2.71	57.1	0.04	0.65	0.37	0.32	1.00
Core B.13	283	0.03	36.7	1.18	0.29	0.37	0.32	256	27.1	50.8	3.29	86.1	0.08	1.46	0.47	0.34	1.74
Core B.14	178	0.09	33.9	0.61	0.18	0.24	0.25	182	19.7	37.9	2.66	62.7	0.04	0.67	0.38	0.32	1.15
Core B.15	280	0.07	36.9	1.09	0.26	0.32	0.29	263	25.3	46.3	2.99	80.5	0.06	1.14	0.43	0.42	1.24
Core B.16	187	0.09	52.0	0.48	0.17	0.26	0.37	199	21.1	41.0	2.71	68.0	0.05	0.77	0.56	0.27	1.51
Core B.17	285	0.06	52.5	1.11	0.26	0.37	0.37	276	24.9	48.4	3.42	75.2	0.07	1.12	0.61	0.48	1.20
Core B.18	192	0.13	38.8	0.53	0.17	0.25	0.25	194	20.0	37.0	2.53	58.9	0.04	0.77	0.40	0.31	1.15
Core B.19	374	0.05	97.3	1.39	0.45	0.55	0.51	372	36.7	71.9	4.58	86.2	0.13	1.59	0.76	0.81	2.20
Core B.20	180	0.08	34.4	0.78	0.19	0.25	0.30	219	21.1	40.2	2.69	66.4	0.05	0.99	0.39	0.34	1.42
Core B.21	401	0.02	47.4	1.36	0.44	0.57	0.39	364	33.8	63.4	4.22	101	0.11	1.44	0.59	0.72	1.62
Core B.22	338	0.03	34.3	0.95	0.30	0.40	0.25	235	26.5	47.7	3.09	75.2	0.09	1.03	0.42	0.42	1.10
Core B.23	525	0.01	32.1	1.32	0.53	0.79	0.27	349	34.3	62.3	3.94	92.1	0.16	1.16	0.48	0.80	0.90
Core B.24	285	0.07	30.9	0.87	0.32	0.41	0.25	288	25.1	49.3	3.18	68.1	0.06	1.10	0.43	0.47	1.05
Core B.25	315	0.05	52.3	0.84	0.34	0.48	0.31	265	24.9	49.1	3.22	65.8	0.08	0.91	0.58	0.53	0.69

**Table 4:** Calculated % relative standard deviation of concentrations in core sediments from site B.

**Sample Label**	**Al**	**Ba**	**Ca**	**Cd**	**Co**	**Cr**	**Cu**	**Fe**	**K**	**Mg**	**Mn**	**Na**	**Ni**	**Pb**	**Sr**	**V**	**Zn**
B1.1	1.03	0.02	0.77	0.22	0.08	0.14	0.03	1.49	3.76	1.59	0.93	1.65	0.02	0.20	0.05	0.14	0.10
B2.1	1.71	0.01	0.86	0.03	0.04	0.11	0.01	1.44	0.98	0.21	0.32	1.74	0.04	0.16	0.16	0.11	0.22
B3.1	1.48	0.03	1.26	0.62	0.03	0.09	0.07	1.43	3.06	0.86	0.64	1.92	0.01	0.11	0.20	0.01	0.72
B4.1	0.75	0.01	0.73	0.63	0.10	0.17	0.05	1.27	0.61	1.77	0.45	1.80	0.03	0.15	0.02	0.36	0.17
B5.1	1.08	0.01	0.64	0.05	0.08	0.09	0.37	2.37	1.43	1.45	0.47	3.39	0.07	0.57	0.90	0.02	0.93
B6.1	2.09	0.01	1.78	0.99	0.11	0.18	0.06	0.93	0.08	0.29	0.14	1.78	0.01	0.98	0.20	0.30	0.17
B7.1	2.68	0.02	0.29	0.63	0.19	0.06	0.19	0.76	1.68	0.58	0.76	2.91	0.03	0.34	0.60	0.15	0.14
B8.1	1.99	0.03	0.45	0.60	0.20	0.31	0.11	0.80	2.25	2.57	1.00	1.14	0.02	0.98	0.08	0.25	0.26
B9.1	2.61	0.06	0.52	0.39	0.04	0.06	0.10	2.05	1.22	0.32	1.48	1.54	0.01	0.30	0.16	0.10	0.38
B10.1	1.72	0.05	0.92	0.32	0.09	0.04	0.05	0.40	1.46	1.52	1.42	2.09	0.04	0.59	0.20	0.27	0.94
B11.1	0.91	0.02	0.95	0.92	0.14	0.06	0.16	2.74	0.55	0.87	1.20	2.39	0.02	0.84	0.10	0.08	0.15
B12.1	1.73	0.02	0.90	0.37	0.04	0.09	0.12	0.79	1.52	0.76	1.36	4.10	0.02	0.18	0.14	0.02	0.14
B13.1	2.76	0.01	2.26	0.62	0.10	0.15	0.11	2.80	1.13	0.30	2.05	0.27	0.01	0.45	0.20	0.12	0.17
B14.1	0.45	0.05	0.76	0.37	0.07	0.11	0.09	1.96	0.53	2.33	0.12	2.77	0.03	0.22	0.12	0.09	0.23
B15.1	2.06	0.01	0.67	0.92	0.11	0.24	0.07	0.79	0.48	0.52	1.36	3.41	0.02	0.18	0.06	0.21	0.24
B16.1	1.16	0.01	0.76	0.29	0.03	0.09	0.05	0.94	1.25	1.48	0.74	3.68	0.01	0.11	0.10	0.07	0.17
B17.1	3.21	0.02	0.65	0.63	0.07	0.03	0.14	0.33	0.38	0.73	1.05	2.29	0.04	0.58	0.07	0.23	0.86
B18.1	0.48	0.09	1.01	0.04	0.01	0.15	0.11	0.89	1.37	0.64	0.50	0.53	0.01	0.21	0.08	0.20	0.21
B.19.1	14.5	0.03	1.41	0.87	0.08	0.09	0.17	2.35	2.01	2.45	0.49	4.54	0.09	0.26	0.07	0.18	0.11
B20.1	1.22	0.02	1.02	0.03	0.02	0.04	0.13	0.65	1.97	1.58	0.20	2.24	0.03	0.22	0.32	0.18	0.18
B21.1	1.67	0.01	1.36	0.97	0.16	0.14	0.30	1.75	0.29	2.02	1.77	1.89	0.06	0.11	0.29	0.65	0.12
B22.1	2.05	0.01	2.85	0.09	0.06	0.17	0.09	0.90	0.57	2.71	2.53	2.14	0.05	0.72	0.15	0.15	0.48
B23.1	0.59	0.01	7.60	0.15	0.17	0.30	0.07	1.31	1.32	0.86	2.94	3.72	0.04	0.13	0.25	0.38	0.11
B24.1	2.70	0.04	1.61	0.11	0.05	0.12	0.13	3.28	1.17	1.27	0.24	1.53	0.03	0.11	0.19	0.22	0.22
B25.1	0.91	0.03	1.09	0.03	0.15	0.22	0.08	1.91	1.07	0.69	2.81	1.53	0.01	0.77	0.21	0.37	0.17

**Table 5:** Concentration (mg/kg) of elements in core sediment samples from site C.

**Sample Label**	**Al**	**Ba**	**Ca**	**Cd**	**Co**	**Cr**	**Cu**	**Fe**	**K**	**Mg**	**Mn**	**Na**	**Ni**	**Pb**	**Sr**	**V**	**Zn**
Core C.1	165	0.15	96.7	0.77	0.13	0.25	0.12	195	17.5	46.5	3.72	38.9	0.03	0.57	0.81	0.28	0.57
Core C.2	265	0.05	40.0	0.87	0.26	0.33	0.17	217	25.2	46.6	3.37	55.2	0.06	0.84	0.45	0.35	0.67
Core C.3	155	0.08	33.4	0.60	0.16	0.22	0.15	168	17.9	34.1	2.49	39.8	0.03	0.34	0.36	0.21	0.68
Core C.4	201	0.07	21.2	0.71	0.16	0.24	0.14	212	19.5	36.3	2.78	39.4	0.04	0.74	0.29	0.30	0.65
Core C.5	298	0.09	39.0	1.10	0.28	0.35	0.21	269	25.8	50.9	3.42	56.0	0.07	1.01	0.46	0.52	0.80
Core C.6	293	0.05	41.9	1.04	0.30	0.40	0.23	241	26.8	51.7	3.42	57.3	0.08	0.94	0.50	0.39	0.95
Core C.7	231	0.05	36.0	0.54	0.24	0.32	0.19	199	23.4	45.2	3.12	52.5	0.06	0.65	0.43	0.35	0.70
Core C.8	224	0.04	45.4	0.88	0.20	0.29	0.19	209	21.3	43.4	3.07	46.7	0.06	0.76	0.48	0.29	0.64
Core C.9	248	0.06	68.9	0.94	0.28	0.37	0.32	248	26.6	55.3	3.43	65.8	0.07	0.91	0.73	0.42	0.84
Core C.10	221	0.10	32.9	0.78	0.26	0.30	0.23	222	24.6	45.6	2.97	55.8	0.05	0.94	0.41	0.41	0.98
Core C.11	234	0.10	42.6	0.74	0.25	0.34	0.54	214	23.6	43.5	2.77	76.7	0.08	1.12	0.48	0.36	2.24
Core C.12	217	0.06	45.2	0.83	0.24	0.29	0.38	212	22.1	41.8	2.84	63.2	0.05	0.93	0.53	0.36	1.31
Core C.13	317	0.05	38.0	1.11	0.33	0.40	0.46	299	28.6	52.9	3.44	93.7	0.09	1.30	0.50	0.57	1.90
Core C.14	230	0.08	36.6	0.57	0.26	0.31	0.37	234	23.8	45.2	2.80	72.2	0.06	1.04	0.45	0.40	1.55
Core C.15	354	0.02	34.2	1.20	0.35	0.45	0.46	268	28.4	49.2	3.13	88.8	0.10	1.21	0.46	0.48	1.86
Core C.16	393	0.02	86.7	1.49	0.42	0.54	0.57	336	34.6	63.8	3.94	111	0.13	1.57	0.89	0.61	2.01
Core C.17	286	0.03	33.6	0.92	0.28	0.36	0.28	240	25.3	45.5	3.02	71.4	0.07	0.98	0.43	0.40	1.24
Core C.18	342	0.06	36.8	1.25	0.36	0.44	0.62	317	29.7	50.3	3.02	100	0.11	1.43	0.49	0.57	2.62
Core C.19	248	0.08	39.2	0.65	0.24	0.31	0.41	196	22.5	39.4	2.34	76.3	0.07	0.78	0.41	0.29	1.76
Core C.20	309	0.03	64.5	0.35	0.37	0.44	0.82	275	27.6	53.5	3.20	99.4	0.11	1.68	0.73	0.38	2.85
Core C.21	140	0.12	33.6	0.42	0.13	0.22	0.38	124	15.6	28.8	1.91	56.6	0.04	0.44	0.35	0.20	1.71
Core C.22	274	0.07	47.6	1.00	0.27	0.41	0.69	247	24.6	47.0	2.60	98.1	0.09	1.38	0.56	0.40	2.89
Core C.23	356	0.05	64.2	1.12	0.41	0.58	1.10	387	36.8	77.3	4.20	167.4	0.15	2.00	0.84	0.80	4.20
Core C.24	114	0.11	37.5	0.50	0.08	0.20	0.37	139	15.0	29.1	2.25	52.2	0.03	0.50	0.40	0.21	1.66
Core C.25	200	0.06	59.5	0.84	0.14	0.37	0.80	195	20.4	45.6	3.02	96.8	0.09	0.88	0.73	0.27	2.82

**Table 6:** Calculated % relative standard deviation of concentrations in core sediments from site C.

**Sample Label**	**Al**	**Ba**	**Ca**	**Cd**	**Co**	**Cr**	**Cu**	**Fe**	**K**	**Mg**	**Mn**	**Na**	**Ni**	**Pb**	**Sr**	**V**	**Zn**
C1.1	4.32	0.08	1.58	0.43	0.09	0.05	0.07	0.84	0.11	1.47	1.17	2.92	0.01	0.19	0.57	0.04	0.27
C2.1	1.48	0.02	1.24	0.39	0.17	0.02	0.06	1.42	2.71	0.61	1.56	2.68	0.02	0.26	0.22	0.05	0.15
C3.1	0.84	0.06	0.12	0.30	0.04	0.06	0.10	1.45	0.54	0.60	1.29	2.55	0.01	0.07	0.22	0.07	0.20
C4.1	1.10	0.03	0.59	0.24	0.09	0.03	0.03	0.82	0.94	0.96	1.11	2.44	0.02	0.08	0.07	0.04	0.12
C5.1	0.53	0.05	1.25	0.52	0.11	0.05	0.01	0.99	1.58	1.02	1.17	1.25	0.03	0.55	0.17	0.07	0.20
C6.1	1.67	0.01	1.27	0.12	0.26	0.03	0.09	0.28	1.35	0.56	0.62	0.98	0.03	0.13	0.09	0.04	0.13
C7.1	2.37	0.01	1.42	0.38	0.07	0.16	0.02	1.93	0.96	0.38	1.33	1.89	0.01	0.11	0.09	0.08	0.17
C8.1	2.98	0.02	0.78	0.30	0.12	0.15	0.12	0.78	1.04	0.71	0.93	1.31	0.03	0.22	0.10	0.02	0.28
C9.1	0.76	0.01	0.48	0.27	0.15	0.10	0.09	0.46	1.67	0.88	1.10	1.81	0.01	0.24	0.12	0.08	0.25
C10.1	0.32	0.05	1.36	0.35	0.11	0.13	0.09	2.16	1.96	1.24	1.48	1.07	0.02	0.10	0.21	0.08	0.22
C11.1	1.34	0.09	1.34	0.31	0.50	0.12	0.11	0.99	0.77	0.15	0.34	1.44	0.03	0.11	0.29	0.05	0.21
C12.1	1.72	0.01	0.96	0.14	0.12	0.09	0.19	1.23	1.46	1.03	1.58	1.46	0.02	0.67	0.12	0.04	0.11
C13.1	0.89	0.03	1.13	0.19	0.16	0.08	0.06	0.45	0.38	1.34	0.87	4.01	0.01	0.17	0.05	0.08	0.64
C14.1	0.47	0.01	1.02	0.32	0.12	0.24	0.07	1.73	1.22	1.95	1.89	0.22	0.03	0.46	0.04	0.09	1.06
C15.1	1.71	0.01	0.44	0.22	0.06	0.25	0.05	0.90	0.42	1.63	1.07	0.52	0.03	0.41	0.18	0.04	0.41
C16.1	1.08	0.01	0.48	0.10	0.03	0.13	0.17	0.30	3.24	0.35	1.69	0.39	0.08	0.48	0.58	0.04	0.12
C17.1	0.89	0.01	1.00	0.72	0.05	0.14	0.12	1.46	1.44	1.87	0.62	1.52	0.04	0.08	0.12	0.04	0.23
C18.1	2.42	0.02	0.18	0.23	0.15	0.25	0.06	1.54	3.14	1.21	1.82	4.39	0.08	0.21	0.05	0.14	1.00
C19.1	1.74	0.01	0.03	0.30	0.20	0.16	0.26	0.57	0.57	1.30	0.78	1.82	0.05	0.17	0.05	0.18	0.73
C20.1	0.63	0.01	1.64	0.10	0.13	0.24	0.11	0.56	1.01	1.99	1.71	0.79	0.02	0.16	0.13	0.04	0.16
C21.1	2.62	0.01	1.73	0.19	0.12	0.02	0.11	1.24	1.45	0.99	0.48	1.47	0.01	0.08	0.08	0.12	0.15
C22.1	2.91	0.01	1.46	0.03	0.09	0.05	0.12	1.83	0.97	0.91	0.50	1.20	0.01	0.83	0.14	0.03	0.17
C23.1	1.05	0.01	1.56	0.26	0.26	0.11	0.62	2.63	0.74	0.69	0.90	2.64	0.01	0.67	0.44	0.06	0.14
C24.1	0.35	0.02	0.88	0.02	0.09	0.02	0.04	1.40	1.36	0.68	0.44	2.28	0.01	0.25	0.17	0.02	0.21
C25.1	2.28	0.01	0.81	0.26	0.01	0.18	0.11	1.77	1.23	0.50	0.63	0.18	0.02	0.34	0.08	0.07	0.14

**Table 7:** Concentration (mg/kg) of elements in core sediment samples from site D.

**Sample Label**	**Al**	**Ba**	**Ca**	**Cd**	**Co**	**Cr**	**Cu**	**Fe**	**K**	**Mg**	**Mn**	**Na**	**Ni**	**Pb**	**Sr**	**V**	**Zn**
Core D.1	131	0.10	36.7	0.61	0.11	0.27	0.74	171	17.1	32.3	2.13	54.7	0.06	1.21	0.40	0.30	3.62
Core D.2	225	0.04	43.8	0.90	0.20	0.39	0.90	226	22.0	40.8	2.50	68.2	0.09	1.93	0.53	0.37	4.24
Core D.3	156	0.06	39.4	0.81	0.18	0.31	0.74	198	21.4	35.4	2.42	49.4	0.07	1.31	0.43	0.31	3.14
Core D.4	293	0.04	38.3	1.21	0.39	0.48	1.01	285	28.9	47.6	2.86	72.2	0.11	2.36	0.49	0.55	4.27
Core D.5	180	0.67	32.5	0.70	0.22	0.31	0.67	181	20.0	35.0	2.24	52.3	0.06	1.06	0.36	0.35	2.95
Core D.6	320	0.05	45.7	1.04	0.34	0.44	0.64	246	26.9	45.9	2.65	67.8	0.11	1.57	0.47	0.44	2.63
Core D.7	282	0.06	49.8	1.06	0.36	0.45	0.87	268	28.9	48.7	2.82	71.7	0.11	1.91	0.53	0.51	3.52
Core D.8	159	0.04	102	0.96	0.16	0.37	0.37	168	23.2	43.0	3.27	112	0.05	0.72	0.92	0.13	3.77
Core D.9	247	0.06	78.4	0.78	0.30	0.35	0.59	235	25.6	43.7	2.71	62.2	0.08	1.47	0.67	0.45	2.46
Core D.10	144	0.08	69.9	0.80	0.17	0.26	0.63	187	18.3	32.4	2.10	44.6	0.05	0.96	0.61	0.29	2.96
Core D.11	208	0.05	53.5	1.01	0.26	0.35	0.82	231	24.6	42.7	2.81	53.5	0.09	1.65	0.58	0.38	3.32
Core D.12	336	0.03	98.0	1.48	0.45	0.54	1.17	352	36.6	63.4	3.76	82.4	0.13	2.08	0.97	0.81	4.18
Core D.13	118	0.09	31.4	0.54	0.16	0.23	0.63	168	17.3	27.9	2.15	34.3	0.04	1.57	0.32	0.33	3.14
Core D.14	291	0.06	54.4	1.15	0.43	0.43	0.81	297	28.5	54.0	3.22	64.1	0.11	2.26	0.62	0.66	3.59
Core D.15	290	0.05	60.2	0.90	0.33	0.40	0.61	235	26.4	45.0	2.69	66.5	0.09	1.51	0.56	0.47	2.59
Core D.16	295	0.04	98.1	1.36	0.33	0.45	0.53	262	26.1	45.6	3.42	46.6	0.09	1.59	0.90	0.43	2.65
Core D.17	371	0.44	93.3	1.54	0.42	0.55	0.63	332	31.2	90.3	3.87	63.0	0.11	1.94	0.77	0.71	3.02
Core D.18	240	0.07	78.2	0.95	0.30	0.40	0.45	262	25.3	43.7	3.22	42.2	0.09	1.66	0.63	0.54	3.14
Core D.19	304	0.05	77.7	1.22	0.40	0.49	0.55	330	28.9	51.0	3.52	46.8	0.11	1.87	0.75	0.64	3.33
Core D.20	289	0.04	80.3	1.30	0.42	0.46	0.45	314	27.1	50.2	3.32	42.3	0.08	1.72	0.71	0.63	2.16
Core D.21	247	0.05	75.4	1.02	0.35	0.40	0.47	253	24.0	43.8	3.02	33.1	0.07	1.52	0.64	0.52	2.60
Core D.22	337	0.03	67.0	0.98	0.44	0.53	0.47	339	31.2	52.8	3.55	42.8	0.11	1.75	0.62	0.70	2.42
Core D.23	204	0.05	47.6	0.62	0.28	0.36	0.39	184	17.2	40.3	3.68	30.1	0.06	0.68	0.91	0.48	2.05
Core D.24	276	0.08	42.2	0.78	0.32	0.42	0.29	223	19.8	34.6	2.37	27.7	0.05	1.06	0.42	0.50	1.08
Core D.25	240	0.03	78.6	0.94	0.37	0.42	0.29	193	19.1	38.4	2.99	28.7	0.07	0.94	0.87	0.42	1.43

**Table 8:** Calculated % relative standard deviation of concentrations in core sediments from site D.

**Sample Label**	**Al**	**Ba**	**Ca**	**Cd**	**Co**	**Cr**	**Cu**	**Fe**	**K**	**Mg**	**Mn**	**Na**	**Ni**	**Pb**	**Sr**	**V**	**Zn**
D1.1	1.51	0.04	1.37	0.23	0.03	0.15	0.07	1.50	0.88	1.48	0.32	2.29	0.02	0.25	0.17	0.11	0.62
D2.1	1.98	0.01	1.35	0.12	0.13	0.11	0.06	1.07	0.30	1.83	0.17	1.64	0.03	0.75	0.24	0.04	0.43
D3.1	0.42	0.02	1.09	0.19	0.10	0.09	0.08	0.76	1.37	1.21	0.21	1.97	0.02	0.12	0.07	0.10	0.96
D4.1	3.88	0.02	0.86	0.32	0.13	0.13	0.17	0.40	0.98	0.96	0.14	0.64	0.02	0.31	0.17	0.08	0.56
D5.1	0.38	0.14	0.64	0.29	0.20	0.29	0.20	0.37	0.64	2.18	0.32	2.46	0.01	0.78	0.15	0.06	0.71
D6.1	3.60	0.02	0.64	0.36	0.16	0.24	0.10	0.65	1.86	1.31	0.42	0.81	0.02	0.86	0.05	0.06	0.77
D7.1	2.57	0.01	0.74	1.40	0.08	0.27	0.13	1.83	1.02	1.03	0.84	1.10	0.02	0.68	0.07	0.38	0.55
D8.1	0.93	0.02	1.37	0.46	0.10	0.05	0.10	0.70	1.64	0.56	0.19	3.20	0.02	0.42	0.08	0.06	0.31
D9.1	0.35	0.04	0.94	0.54	0.13	0.22	0.33	1.10	0.76	3.00	0.58	1.34	0.03	0.14	0.24	0.17	0.19
D10.1	1.53	0.01	1.95	0.30	0.07	0.09	0.47	1.06	0.25	0.54	0.11	0.95	0.01	0.20	0.49	0.14	0.16
D11.1	0.38	0.03	0.58	0.43	0.10	0.02	0.08	0.76	0.65	1.34	0.87	2.05	0.04	0.23	0.12	0.12	0.13
D12.1	0.44	0.01	0.37	0.19	0.08	0.20	0.14	1.21	2.36	2.19	0.22	0.73	0.01	0.32	0.12	0.07	0.21
D13.1	1.33	0.05	1.42	0.16	0.12	0.03	0.08	0.60	0.84	1.26	0.20	1.20	0.03	0.77	0.05	0.04	0.17
D14.1	2.58	0.03	1.41	0.27	0.13	0.08	0.09	0.84	1.80	2.08	0.18	3.93	0.01	0.95	0.08	0.05	0.23
D15.1	2.97	0.02	1.46	0.31	0.08	0.12	0.32	0.72	1.47	1.74	0.63	2.63	0.05	0.79	0.05	0.05	0.16
D16.1	0.87	0.01	0.79	0.26	0.14	0.26	0.17	0.44	1.41	1.25	0.42	2.78	0.02	0.52	0.11	0.02	0.22
D17.1	1.06	0.03	2.22	0.24	0.08	0.29	0.06	0.25	1.22	0.62	0.27	6.02	0.07	0.56	0.36	0.04	0.17
D18.1	0.19	0.01	1.51	0.31	0.12	0.24	0.14	0.53	0.22	0.87	0.31	0.82	0.01	0.15	0.14	0.09	0.19
D19.1	1.45	0.02	0.48	0.38	0.15	0.26	0.19	1.96	0.85	0.64	0.20	1.68	0.02	0.29	0.04	0.08	0.28
D20.1	0.77	0.02	0.92	0.34	0.08	0.11	0.10	1.72	0.26	2.36	0.12	1.67	0.01	0.18	0.15	0.06	0.20
D21.1	1.50	0.02	0.72	0.52	0.11	0.12	0.10	0.77	2.12	1.86	0.45	1.05	0.04	0.11	0.14	0.23	0.21
D22.1	0.26	0.01	0.19	0.38	0.04	0.19	0.10	1.00	1.22	0.66	0.17	5.23	0.01	0.74	0.20	0.12	0.16
D23.1	1.48	0.03	1.90	0.30	0.02	0.09	0.25	0.63	0.68	1.38	0.22	0.47	0.04	0.32	0.15	0.15	0.18
D24.1	2.03	0.02	1.62	0.07	0.03	0.04	0.01	1.58	0.97	0.46	0.33	1.81	0.03	0.11	0.05	0.10	0.27
D25.1	0.74	0.01	1.20	0.18	0.03	0.03	0.13	0.48	1.27	2.97	0.58	2.98	0.06	0.12	0.07	0.13	0.23

**Table 9:** Hazard index calculated for heavy metals in core sediment samples from Lagos lagoon.

	**Cd**	**Cr**	**Cu**	**Ni**	**Pb**	**Zn**		**Cd**	**Cr**	**Cu**	**Ni**	**Pb**	**Zn**
Core A.1	0.66	0.02	0.01	0.00	0.01	0.01	Core B.1	1.18	0.01	0.01	0.00	0.02	0.01
Core A.2	1.30	0.03	0.01	0.00	0.02	0.02	Core B.2	1.72	0.01	0.01	0.00	0.04	0.02
Core A.3	1.37	0.04	0.02	0.00	0.03	0.02	Core B.3	1.68	0.01	0.01	0.00	0.02	0.01
Core A.4	1.58	0.04	0.02	0.00	0.04	0.02	Core B.4	1.48	0.01	0.01	0.00	0.03	0.01
Core A.5	3.38	0.09	0.05	0.01	0.09	0.05	Core B.5	1.98	0.01	0.01	0.01	0.05	0.01
Core A.6	1.58	0.04	0.02	0.00	0.03	0.03	Core B.6	2.45	0.01	0.01	0.01	0.04	0.02
Core A.7	1.07	0.03	0.02	0.00	0.03	0.02	Core B.7	1.76	0.01	0.01	0.00	0.04	0.01
Core A.8	1.37	0.04	0.01	0.00	0.01	0.02	Core B.8	2.16	0.01	0.01	0.01	0.03	0.01
Core A.9	2.60	0.07	0.03	0.01	0.04	0.02	Core B.9	0.89	0.01	0.01	0.00	0.03	0.01
Core A.10	1.90	0.05	0.02	0.00	0.03	0.02	Core B.10	1.68	0.01	0.01	0.00	0.03	0.01
Core A.11	0.86	0.02	0.01	0.00	0.01	0.02	Core B.11	2.00	0.01	0.01	0.01	0.04	0.01
Core A.12	1.11	0.03	0.02	0.00	0.02	0.02	Core B.12	0.88	0.01	0.01	0.00	0.02	0.01
Core A.13	0.59	0.02	0.02	0.00	0.02	0.02	Core B.13	1.98	0.01	0.01	0.00	0.04	0.01
Core A.14	1.05	0.03	0.02	0.00	0.03	0.02	Core B.14	1.02	0.01	0.01	0.00	0.02	0.01
Core A.15	1.34	0.04	0.02	0.00	0.03	0.02	Core B.15	1.83	0.01	0.01	0.00	0.03	0.01
Core A.16	1.42	0.04	0.02	0.00	0.02	0.02	Core B.16	0.80	0.01	0.01	0.00	0.02	0.01
Core A.17	0.95	0.03	0.02	0.00	0.03	0.02	Core B.17	1.86	0.01	0.01	0.00	0.03	0.01
Core A.18	0.90	0.02	0.02	0.00	0.02	0.02	Core B.18	0.89	0.01	0.01	0.00	0.02	0.01
Core A.19	2.15	0.06	0.03	0.01	0.06	0.03	Core B.19	2.33	0.01	0.01	0.01	0.05	0.02
Core A.20	1.03	0.03	0.02	0.01	0.04	0.02	Core B.20	1.32	0.01	0.01	0.00	0.03	0.01
Core A.21	1.56	0.04	0.02	0.00	0.04	0.02	Core B.21	2.28	0.01	0.01	0.01	0.04	0.01
Core A.22	1.28	0.03	0.01	0.00	0.02	0.02	Core B.22	1.6.0	0.01	0.01	0.01	0.03	0.01
Core A.23	1.03	0.03	0.01	0.00	0.02	0.02	Core B.23	2.22	0.02	0.01	0.01	0.03	0.01
Core A.24	1.56	0.04	0.01	0.00	0.03	0.01	Core B.24	1.47	0.01	0.01	0.00	0.03	0.01
Core A.25	0.98	0.03	0.01	0.00	0.02	0.01	Core B.25	1.40	0.01	0.01	0.00	0.03	0.01
***m*****HQ**	0.85	0.01	0.01	0.00	0.02	0.02		1.00	0.01	0.01	0.00	0.02	0.01
Core C.1	1.30	0.01	0.00	0.00	0.02	0.00	Core D.1	1.02	0.01	0.02	0.00	0.03	0.03
Core C.2	1.46	0.01	0.00	0.00	0.02	0.01	Core D.2	1.51	0.01	0.03	0.01	0.06	0.03
Core C.3	1.01	0.01	0.00	0.00	0.01	0.01	Core D.3	1.36	0.01	0.02	0.00	0.04	0.03
Core C.4	1.19	0.01	0.00	0.00	0.02	0.01	Core D.4	2.03	0.01	0.03	0.01	0.07	0.03
Core C.5	1.85	0.01	0.01	0.00	0.03	0.01	Core D.5	1.18	0.01	0.02	0.00	0.03	0.02
Core C.6	1.75	0.01	0.01	0.00	0.03	0.01	Core D.6	1.75	0.01	0.02	0.01	0.04	0.02
Core C.7	0.91	0.01	0.01	0.00	0.02	0.01	Core D.7	1.78	0.01	0.02	0.01	0.05	0.03
Core C.8	1.48	0.01	0.01	0.00	0.02	0.01	Core D.8	1.61	0.01	0.01	0.00	0.02	0.03
Core C.9	1.57	0.01	0.01	0.00	0.03	0.01	Core D.9	1.30	0.01	0.02	0.00	0.04	0.02
Core C.10	1.31	0.01	0.01	0.00	0.03	0.01	Core D.10	1.33	0.01	0.02	0.00	0.03	0.02
Core C.11	1.24	0.01	0.02	0.00	0.03	0.02	Core D.11	1.70	0.01	0.02	0.01	0.05	0.03
Core C.12	1.39	0.01	0.01	0.00	0.03	0.01	Core D.12	2.48	0.01	0.03	0.01	0.06	0.03
Core C.13	1.86	0.01	0.01	0.00	0.03	0.02	Core D.13	0.90	0.01	0.02	0.00	0.04	0.03
Core C.14	0.95	0.01	0.01	0.00	0.03	0.01	Core D.14	1.93	0.01	0.02	0.01	0.06	0.03
Core C.15	2.02	0.01	0.01	0.00	0.03	0.02	Core D.15	1.50	0.01	0.02	0.01	0.04	0.02
Core C.16	2.50	0.01	0.02	0.01	0.04	0.02	Core D.16	2.29	0.01	0.01	0.01	0.05	0.02
Core C.17	1.54	0.01	0.01	0.00	0.03	0.01	Core D.17	2.59	0.01	0.02	0.01	0.06	0.02
Core C.18	2.10	0.01	0.02	0.01	0.04	0.02	Core D.18	1.59	0.01	0.01	0.01	0.05	0.03
Core C.19	1.09	0.01	0.01	0.01	0.02	0.01	Core D.19	2.05	0.01	0.02	0.01	0.05	0.03
Core C.20	0.58	0.01	0.02	0.00	0.05	0.02	Core D.20	2.18	0.01	0.01	0.01	0.05	0.02
Core C.21	0.69	0.01	0.01	0.01	0.01	0.01	Core D.21	1.71	0.01	0.01	0.00	0.04	0.02
Core C.22	1.67	0.01	0.02	0.01	0.04	0.02	Core D.22	1.64	0.01	0.01	0.01	0.05	0.02
Core C.23	1.88	0.01	0.03	0.00	0.06	0.03	Core D.23	1.05	0.01	0.01	0.00	0.02	0.02
Core C.24	0.83	0.01	0.01	0.00	0.01	0.01	Core D.24	1.31	0.01	0.01	0.00	0.03	0.01
Core C.25	1.42	0.01	0.02	0.00	0.03	0.02	Core D.25	1.57	0.01	0.01	0.00	0.03	0.01
***m*****HQ**	0.87	0.01	0.01	0.00	0.02	0.01		1.02	0.01	0.01	0.00	0.03	0.02

## 2 Experimental design, materials and methods

A total of one hundred (100) core sediment samples were collected using a 50 cm×3.5 cm Wildco® hand corer, and stored in labelled plastic Ziploc bags and kept at 4 °C prior to transportation to the laboratory. Samples were collected from four locations and each sampling location was geo-referenced using a handheld Garmin GPS. The sediment sub-samples were recovered at every 2 cm interval of the core. Samples were kept frozen until analyzed. The samples were oven-dried at 50 °C and 0.5 g of air-dried sediment samples were accurately weighed and passed through 2 mm sieve. Wet digestion procedure using acid mixture of HClO_4_/HNO_3_/H_2_SO_4_ was used for total digestion of sediment samples before elemental analysis using Agilent 4200 Microwave Plasma Atomic Emission Spectrometry (MP-AES). All elemental determinations were carried out in triplicate and averaged with measurement errors < 5%. The accuracy and precision of each batch of analytical procedure were routinely evaluated using certified standard reference material and standard spiked samples. The accepted recoveries ranged from 75% to 108%. The estimation of the degree of heavy metal contamination was assessed using pollution indexes including hazard quotient (HQ) [1], [2], modified hazard quotient (*m*HQ) [1], [3]. The sediment quality guidelines adopted for calculating the HQ and *m*HQ were the threshold effects level (TEL), probable effect level (PEL), and severity effect level (SEL), [4], [5], with classifications as proposed by Feng et al. [6]. The classification of the degree of sediment contamination by trace metals was done as proposed by Benson et al. [3].
